# Replica of Bionic Nepenthes Peristome-like and Anti-Fouling Structures for Self-Driving Water and Raman-Enhancing Detection

**DOI:** 10.3390/polym14122465

**Published:** 2022-06-17

**Authors:** Yen-Ting Lin, Chun-Hao Wu, Wei-Lin Syu, Po-Cheng Ho, Zi-Ling Tseng, Ming-Chien Yang, Chin-Ching Lin, Cheng-Chen Chen, Cheng-Cheung Chen, Ting-Yu Liu

**Affiliations:** 1 Department of Materials Engineering, Ming Chi University of Technology, New Taipei City 243303, Taiwan; ken851028@gmail.com (Y.-T.L.); m10188005@mail2.mcut.edu.tw (C.-H.W.); shewinnie43@gmail.com (W.-L.S.); gf183183@gmail.com (P.-C.H.); 2 Department of Materials Science and Engineering, National Taiwan University of Science and Technology, Taipei 106335, Taiwan; grace5032731@gmail.com (Z.-L.T.); myang@mail.ntust.edu.tw (M.-C.Y.); 3 Senguan Tech Co., Ltd., Tainan City 71076, Taiwan; chinchinglin@hotmail.com; 4 Department of Architecture, National Taipei University of Technology, Taipei 106344, Taiwan; 5 Institute of Preventive Medicine, National Defense Medical Center, New Taipei City 23742, Taiwan; 6 Graduate Institute of Medical Science, National Defense Medical Center, Taipei 11490, Taiwan

**Keywords:** Nepenthes structure, bionic replica, zwitterion polymers, self-driving water, unidirectional water delivery, surface-enhanced Raman scattering (SERS) detection

## Abstract

The flexible, anti-fouling, and bionic surface-enhanced Raman scattering (SERS) biochip, which has a Nepenthes peristome-like structure, was fabricated by photolithography, replicated technology, and thermal evaporation. The pattern of the bionic Nepenthes peristome-like structure was fabricated by two layers of photolithography with SU-8 photoresist. The bionic structure was then replicated by polydimethylsiloxane (PDMS) and grafting the zwitterion polymers (2-methacryloyloxyethyl phosphorylcholine, MPC) by atmospheric plasma polymerization (PDMS-PMPC). The phospholipid monomer of MPC immobilization plays an important role; it can not only improve hydrophilicity, anti-fouling and anti-bacterial properties, and biocompatibility, but it also allows for self-driving and unidirectional water delivery. Ag nanofilms (5 nm) were deposited on a PDMS (PDMS-Ag) substrate by thermal evaporation for SERS detection. Characterizations of the bionic SERS chips were measured by a scanning electron microscope (SEM), optical microscope (OM), X-ray photoelectron spectrometer (XPS), Fourier-transform infrared spectroscopy (FTIR), and contact angle (CA) testing. The results show that the superior anti-fouling capability of proteins and bacteria (*E. coli*) was found on the PDMS-PMPC substrate. Furthermore, the one-way liquid transfer capability of the bionic SERS chip was successfully demonstrated, which provides for the ability to separate samples during the flow channel, and which was detected by Raman spectroscopy. The SERS intensity (adenine, 10^−4^ M) of PDMS-Ag with a bionic structure is ~4 times higher than PDMS-Ag without a bionic structure, due to the multi-reflection of the 3D bionic structure. The high-sensitivity bionic SERS substrate, with its self-driving water capability, has potential for biomolecule separation and detection.

## 1. Introduction

There are many micro- or nano-scale structures with different functions and special characteristics in nature, which have not yet been discovered because of the limitations of existing detection technologies. A benefit of the progress of manufacturing and the advancement in detection techniques in recent years has been that the micro-scale structure can now easily be observed, encouraging more researchers to invest in micro biomaterial structures. For example, lotus leaves possess a super-hydrophobic and self-cleaning ability due to the unique nanostructure of their surface [1,2]. This nanostructure contains structural wax on the surface layer, which not only increases surface roughness, but also traps air between the solid and liquid interface [3]. Biomimetic technology [4,5,6,7,8,9,10,11,12] is a technology that imitates the 3.8 billion years of evolutionary experience of living things. Every lifestyle, growth process, and ecosystem is a source of inspiration for simulation. It has the two major contradictions of “nature but primitive” and “technology but pollution”, which are currently significant [13]. Biomimetic technology has three advantages: low costs, high efficiency, and low pollution. With the proper utilization of this technology, we can achieve low resource and energy consumption while obtaining optimal production capacity and benefits. For example, aircraft coatings based on shark skin resistance [6,14,15,16], radar sonar based on bats [17], solar applications inspired by photosynthesis, bullet train heads based on kingfisher beaks [18,19], antifouling coatings based on frog skins [20], etc. Biomaterials have also been widely applied in SERS substrates due to their 3D periodic microstructures, such as butterfly wings, cicada wings, and rose petals [21,22,23]. The best case for the continuation, this study focuses on the surface structure of nepenthes.

Nepenthes lives in a barren environment. Uniquely, they can capture insects with their peristome to meet their fundamental nutrient needs [2]. Most species obtain their nutrients from trapped and food animals. The inner surface of the insect trap is covered with slippery and fragile wax [24], forming an effective trap. The peristome on the top aims to attract and capture prey by forming a smooth liquid film. Previous studies indicated that the peristome structure of Nepenthes consists of radially arranged ribs. Rain, dew, and honey form a layer of liquid film on the surface, transforming the peristome in a super-hydrophilic surface. The surface of the epidermal cells of Nepenthes peristome is smooth and wax-free. The addition of wax-free crystals and hygroscopic honey increases the capillary force and promotes the formation of the liquid film, further enhancing the liquid transport speed on the surface of the Nepenthes peristome [25], which even resists gravity. Nevertheless, the continuous radial arrangement of the peristome grooved structure exhibits liquid transmission characteristics without other external forces. Fabricating a surface with the same structural features as the replica molding method is ideal to achieve the one-way liquid transfer (self-driving water) capability, which has potential to apply in microfluidic devices without pumping. Directly using the natural peristome as a template, the peristome structure can be replicated by artificial poly (dimethylsiloxane) (PDMS); however, the surface is curved and difficult to reprocess. Therefore, we propose a high-precision photolithography process to imitate the Nepenthes peristome.

The plane structure is reproduced on PDMS through transfer-printing technology [13,26,27]. Most studies choose PDMS as the transfer material due to its excellent pattern reproduction accuracy, easy preparation, and ease of observation, etc. A microfluidic chip is a very promising analytical platform for the sample pre-treatment, separation, and detection [28]; for example, a microfluidic based surface-enhanced Raman scattering (SERS) chip was applied to detect creatinine in blood for 2 min [28,29]. The microfluidic SERS chip was proven to effectively detect bio-samples. However, the replica PDMS is an extremely hydrophobic material, whereas Nepenthes peristome has hydrophilic structure; to address this, we propose a hydrophilic modified material with 2-methacryloyloxyethyl phosphorylcholine (MPC), which contains a phospholipid structure that was polymerized and grafted on the PDMS surface by atmospheric plasma. MPC is a zwitterionic monomer with good antithrombotic and blood compatibility [30,31,32]. This polymer is highly hydrophilic due to the phospholipid polar tail in the MPC molecular structure. Moreover, the inhibitory effect on the adhesion of cells, platelets, enzymes, and proteins in the blood can also reduce the chances of the organism recognizing the material as a foreign object, and increases the biocompatibility of the material [33,34]. Therefore, the hydrophilic bionic structure was imitated to exhibit the same liquid transport capability as the Nepenthes peristome in this study.

## 2. Materials and Methods

### 2.1. Photolithography

**Figure 1:**
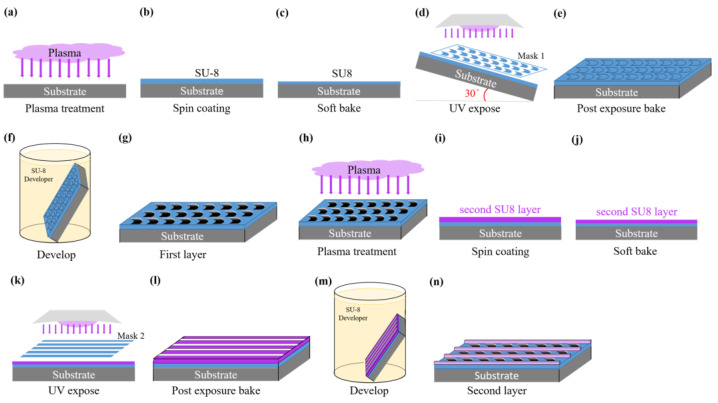
Diagram of the photolithography process of a Nepenthes peristome-like structure.

The photolithography process of a Nepenthes peristome-like structure is shown in Figure 1. The silicon wafer substrate was consecutively cleaned with acetone, ethanol, and deionized water, and cleaned by vacuum plasma for 10 min after being dried by N_2_ gas. After the plasma treatment, the surface achieved temporary hydrophilicity to increase the adhesion of the substrate and photoresist. SU-8 negative photoresist (KAYAKU SU8-2025) was spin-coated at a gradual acceleration speed of 500 rpm for 10 s, 1500 rpm for 10 s, and 3000 rpm for 30 s, which could make the homogenous coating for the SU-8 photoresist. Then, incubation occurred for 30 min before soft baking. Soft baking following gradual heating (65–90 °C) can avoid bubbles caused by an excessive heating rate. SU-8 negative photoresist was cross-linked with a mercury lamp (365 nm) for 25 s, with an exposure process of two layers of photomask patterns. After exposure, adhesion of the exposed pattern and the substrate were improved by post-exposure baking at 90 °C, and then immersed in the developer to remove the un-crosslinked SU-8 photoresist. Following developments, we rinsed with deionized water and hard baked for 3 min to remove the last remaining solvent. The SU-8 mold of the Nepenthes peristome-like structure was then used in the further replica of the PDMS membranes. The easy-cleaning coating solution, Naegix E720 (Senguan Tech Co., Ltd., Tainan City, Taiwan), was used as the control to evaluate the surface energy of a replica of a bionic Nepenthes peristome-like structure.

### 2.2. Replica of a Nepenthes Peristome-like Structure by PDMS

**Figure 2:**
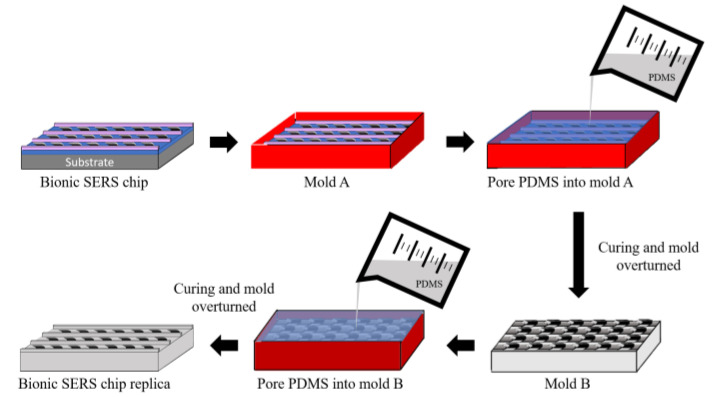
Diagram of the replicated Nepenthes peristome-like structure process by polydimethylsiloxane (PDMS).

Polydimethylsiloxane (PDMS, Dow Corning^®^ Sylgard 184, part A) and a crosslinking agent (Sylgard 184, part B) were mixed with a 10:1 PDMS base to a curing agent ratio. The stiffness of the PDMS can be manipulated by the addition of a curing agent. To avoid bubbles, the mixed PDMS must be placed in a vacuum desiccator until the bubbles disappear. The PDMS solution was then poured into a 35 mm dish and placed in the oven at 60 °C for 4 h until the structure stabilized. SU-8 based bionic (Nepenthes peristome-like) structure was cleaned with deionized water before plasma cleaning for 1 min. Moreover, the platinum was coated on the surface of the SU-8 based bionic structure. After all these steps were completed, the mixed PDMS solution was poured into the SU-8 mold and vacuumed to remove extra bubbles. Then, it stood in the oven at 65 °C for 4 h. The bionic replica of the PDMS membrane was slit to generate the Nepenthes peristome-like structure (Figure 2).

### 2.3. MPC Immobilization by Atmospheric Plasma

**Figure 3:**
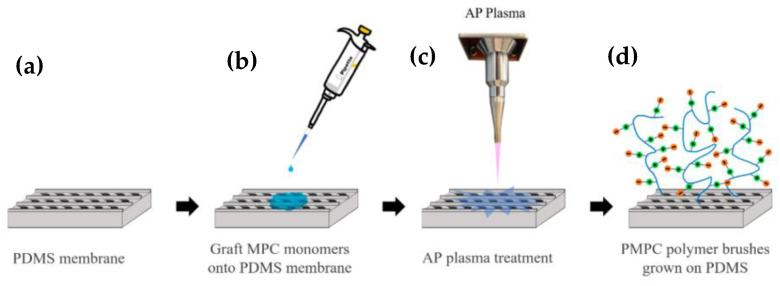
Schematic diagram of the replica of the PDMS-based bionic Nepenthes peristome-like structure with 2-methacryloyloxyethl phosphorylcholine (MPC) immobilization by atmospheric plasma. (**a**) The replica PDMS membrane was pre-treated by plasma cleaning for 1 min; (**b**) PDMS immersed in MPC solution; (**c**) MPC monomers were polymerized by oxygen atmospheric plasma and formed (**d**) MPC polymer brushes.

The zwitterion polymer (2-methacryloyloxyethyl phosphorylcholine, MPC) modification flowchart is shown in Figure 3. The bionic replica of the PDMS membrane was pre-treated by plasma cleaning for 1 min (Figure 3a) and then immersed in MPC solution (Figure 3b) [27,28]. In addition to activating the surface functional groups, the hydrophilic treatment can effectively improve the uniformity of the modification. Subsequently, the pre-treated PDMS was treated by oxygen atmospheric plasma (AP Plasma Jet, Feng Tien Electronic Co., Ltd., Taipei, Taiwan) (Figure 3c) to polymerize the MPC monomers to MPC polymer brushes (Figure 3d) at an operating power of 1.2 kW and oxygen flux rate of 10 slm (L/min) for 10 s, and a 1.2 cm distance was maintained from the plasma torch.

### 2.4. Anti-Bacterial Adhesion Capability

The antibacterial effect of PDMS and PDMS-PMPC against bacteria (*E. coli*) was inspected with the bacterial adhesion method. The samples were shaken with the lysate of *E. coli* at 37 °C. After 24 h, the samples were stained with SYTO 9 (green fluorescence nucleic acid stain) and stood for 5 min. The results were obtained with fluorescence microscope.

### 2.5. Anti-Protein Adhesion Capability

The PDMS and PDMS-PMPC were incubated in a 10 mL phosphate-buffered solution (PBS) solution of albumin from human serum (HSA) in the 24-well tissue culture plate at 37 °C for 1 h. Afterward, the samples were gently washed 3 times using PBS in the 24-well tissue culture plate. Then, the samples were incubated with 1 wt% aqueous solution of sodium dodecyl sulfate (SDS). The BCA kit was used to determine the concentration of the proteins in the SDS solution, and the concentration was detected by UV-vis spectroscopy.

### 2.6. Cell Attachment Tests

The cell attachment was detected with cells adhesion. The PDMS and PDMS-PMPC were placed in the 24-well plate, injected with NIH 3T3 mouse embryonic fibroblast cells (3T3 cells) to the substrate surface, and incubated at 37 °C for 24 h. The cells’ attachment behavior would be evaluated by fluorescent staining (nucleus staining and cell membrane staining) to observe the amount of cell adhesion by fluorescent microscopy.

### 2.7. Biocompatibility

Biocompatibility is defined as the ability of a material to perform with an appropriate host response in a specific application [28]. The biocompatibility of pristine PDMS and PDMS-PMPC was evaluated with the proliferation of 3T3 fibroblast. The substrates (1 cm × 1 cm) and 1 mL solution of 3T3 cells (10^5^ cells/mL) were placed in the 24-well plate under a 5% CO_2_ atmosphere The cell viability was determined at 37 °C for 24, 48, and 72 h by thiazolyl blue tetrazolium bromide (MTT) assay and absorbance at 570 nm.

### 2.8. One-Way Liquid Transfer Capability

One-way liquid transfer capability of PDMS and PDMS-PMPC substrates was measured by the stained deionized water dropped on the PDMS and PDMS-PMPC substrates. The distance of stained deionized water flowing was recorded after 4 min. The flowing distance would be evaluated between pristine PDMS and PDMS-PMPC substrates. The longer flowing distance shows the greater one-way liquid transfer capability.

### 2.9. Characterizations

The morphology of the bionic SERS substrate was observed by scanning electron microscopy (SEM) (JEOL JSM-6701F, Tokyo, Japan). FT-IR spectroscopy (FT-IR, Perkin-Elmer Spectrum-One, Shelton, CT, USA) was used to differentiate the composition and chemical structure of the bionic SERS substrate. The chemical-binding energy of the bionic SERS substrate was carried out by a K-Alpha X-ray photoelectron spectrometer (XPS) (Thermo Fisher Scientific, Waltham, MA, USA). The hydrophilicity of the bionic SERS substrates was recorded by a contact angle goniometer (DSA 100, Krüss GmbH, Hamburg, Germany). Raman spectroscopy (HORIBA, LabRAM HR Evolution) was used to evaluate the SERS spectra of the bionic SERS substrate, with a 632.8 nm He-Ne laser, operated under a 10× objective lens with a detection range of 400–2000 cm^−1^.

## 3. Results and Discussion

### 3.1. Optical Microscope (OM) Analysis

**Figure 4:**
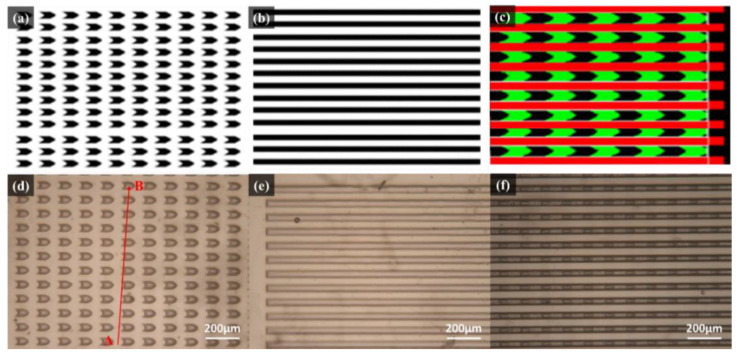
Diagram of (**a**) the first and (**b**) the second layers of photomask patterns; (**c**) overlaid photomask patterns. Optical microscope (OM) image of (**d**) first layer pattern, (**e**) second layer pattern, and (**f**) overlaid layers pattern of SU-8 photoresist. The line point A to point B passes through the plane and the pattern indicates the segmented plane of the cross-section from the first layer of SU-8 photoresist.

**Figure 5:**
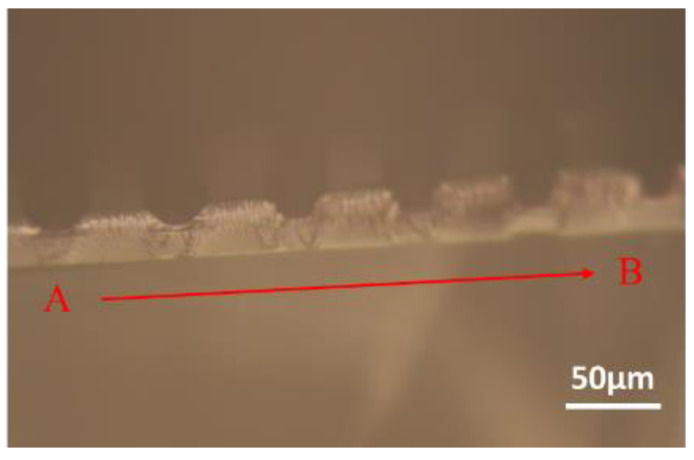
The OM image of the cross-section of the first layer of SU-8 photoresist.

The photomask patterns of the Nepenthes peristome-like structure were shown in Figure 4a–c. The arrow arrays (Figure 4a) were fabricated in the first (bottom) layer. Then, the straight pattern arrays (Figure 4b) were covered as the second layer to develop the bionic Nepenthes peristome-like structure (Figure 4c). Figure 4d–f show the OM images of the first layer (Figure 4d), the second layer (Figure 4e), and the overlaid layers pattern (Figure 4f) of SU-8 photoresist during the photolithographic process. The resulting three patterns are similar to the three photomask patterns. The line point A to point B (Figure 4d and Figure 5) passes through the plane and the pattern represents the segmented plane of the cross-section from the first layer of SU-8 photoresist. Although the arrow array (Figure 4d) becomes broader during the photolithographic process, compared to the pristine photomask patterns (Figure 4a), the gap channels between the arrow arrays do not change too much. Furthermore, the cross-section of the first layer replicated by PDMS was observed in Figure 5. The depth of the gap channel could be clearly observed in the cross-section image, showing the pattern integrity after the photolithographic process.

### 3.2. Scanning Electron Microscope (SEM) Observation

**Figure 6:**
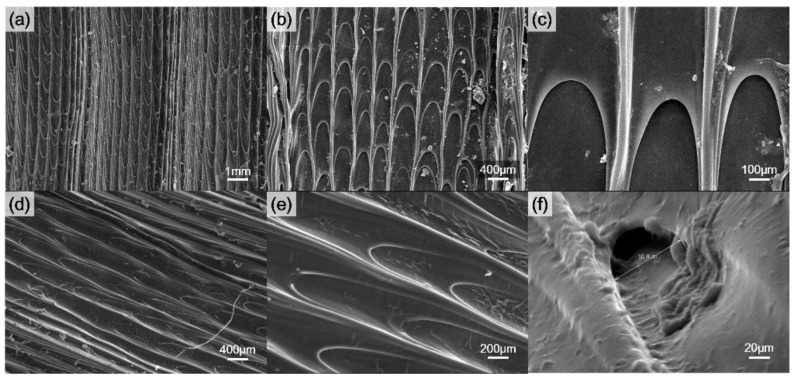
SEM images of (**a**–**c**) the real (pristine) Nepenthes peristome and (**d**–**f**) the replica of the Nepenthes peristome-like structure of PDMS.

From Figure 6a–c, the continuous radially arranged furrows were observed with grooves at the real (pristine) Nepenthes peristome structure. The width was 30–40 µm and the length was 130–160 µm. In comparison, the replica of the PDMS-based bionic Nepenthes peristome-like structure is shown in Figure 6d–f. The double layer structure was successfully fabricated by the replicated process, and the detail structure is very similar to the pristine Nepenthes peristome.

### 3.3. Hydrophilicity by Contact Angle Measurements

**Figure 7:**
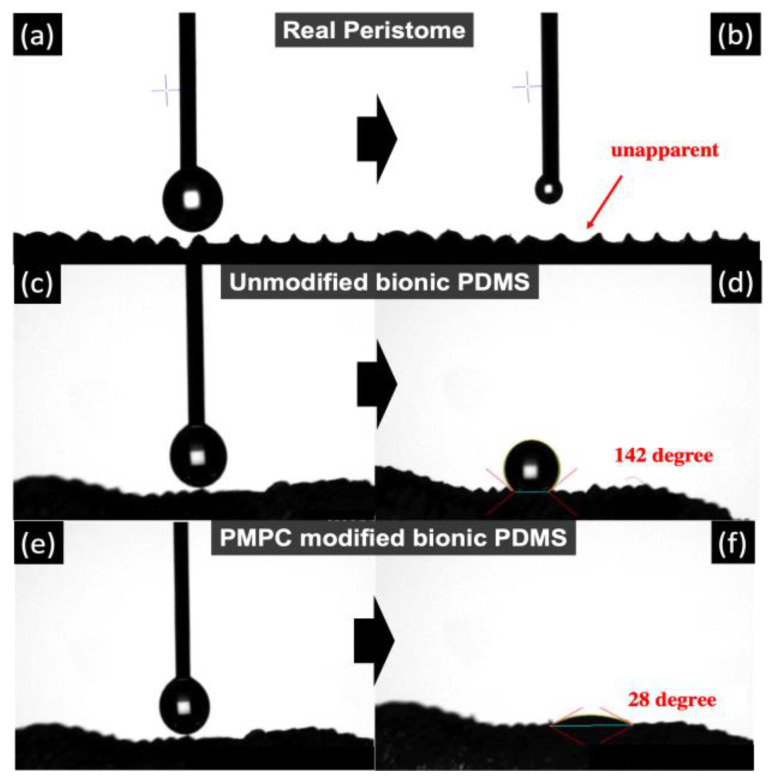
The contact angle of (**a**,**b**) the pristine Nepenthes peristome (almost 0°), (**c**,**d**) Nepenthes peristome-like structure replicated by PDMS without MPC modification, and (**e**,**f**) the Nepenthes peristome-like structure replicated by PDMS with MPC modification.

The contact angle of the pristine Nepenthes peristome exhibiting a super-hydrophilic surface leads to an unapparent water contact angle (almost 0°), as shown in Figure 7a,b. However, the replica of the bionic PDMS surface (without modified MPC) shows the super-hydrophobicity (CA: ~142°) in Figure 7c,d, which is higher than the coating of commercial self-cleaning products (Naegix E720) (~90°). The structure of the replica of bionic PDMS is similar to the pristine Nepenthes peristome, but the surface energy is completely different. The reason for that is because the hydrophilic functional group was found on the pristine Nepenthes peristome, whereas the replica of the bionic PDMS surface is more hydrophobic. Therefore, MPC polymer brushes (PMPC) were grafted on the PDMS substrate by atmospheric plasma to improve hydrophilicity. The contact angle displayed was ~28° after PMPC was immobilized on the PDMS-replicated Nepenthes peristome, which is closer to the pristine Nepenthes peristome (Figure 7e,f).

### 3.4. FTIR Spectrum Analysis

**Figure 8:**
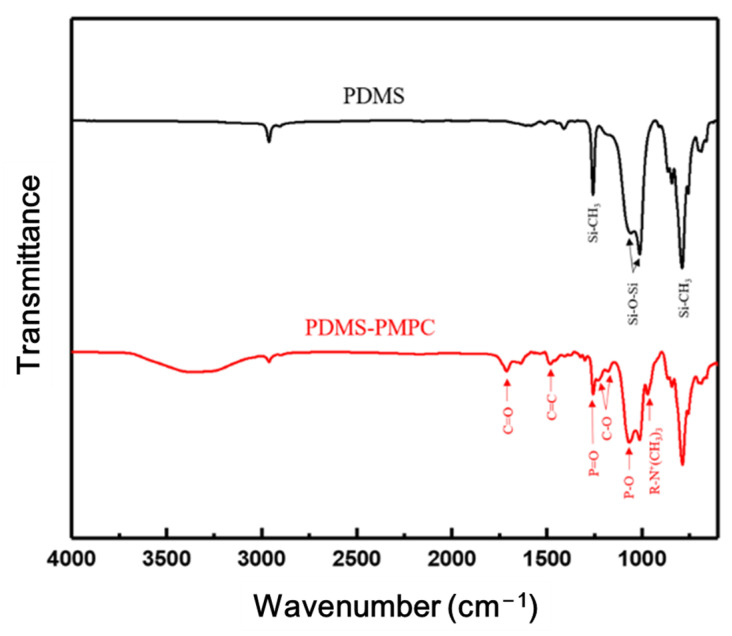
FTIR spectrum of PDMS and MPC polymer brushes (PMPC) were grafted on the PDMS substrate and PDMA-PMPC substrate.

Figure 8 shows the FTIR spectra of pristine PDMS and PDMS coated with PMPC. The FTIR spectrum of PDMS shows the characteristic peaks at a stretching frequency of 789 cm^−^^1^ bending vibration modes of Si-CH_3_ and Si-(CH_2_)_n_-Si, the characteristic peaks at the stretching frequency of 1020 cm^−^^1^ due to the bending vibration modes of the asymmetric Si–O–Si stretching vibrations, and the characteristic peaks at 1259 cm^−^^1^ due to the bending vibration modes of Si-CH_3_ symmetric bending [35,36]. PDMS coated with PMPC has the characteristic peaks of 971 cm^−^^1^, 1090 cm^−^^1^, and 1247 cm^−^^1^ due to P-O, R-N^+^(CH_3_)_3_, and the P=O stretching vibrational band [37,38]. The results confirmed that PMPC polymer brushes were successfully immobilized on the PDMS substrate.

### 3.5. XPS Spectrum Analysis

**Figure 9:**
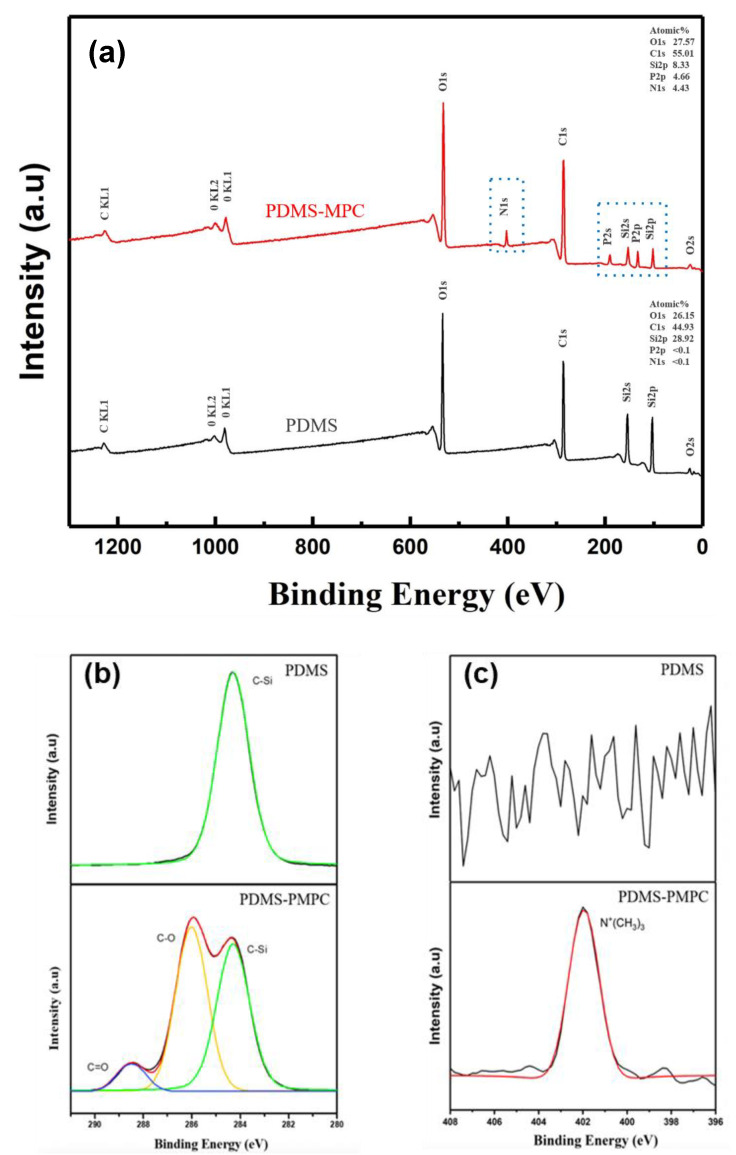
(**a**) XPS full spectra, (**b**) C-1s spectra, and (**c**) N-1s spectra of the PDMS and PDMS-PMPC substrates.

The binding energy change between the PDMS and PDMS-PMPC substrates was measured by an XPS analysis (Figure 9). The full spectra (Figure 9a) show that the surface primarily contained O, Si, C, P, and N. Compared with the PDMS surface, the characteristic peaks of N-1s and P-2s orbitals were observed at the PDMS-PMPC substrates, which contribute to the phosphorus and nitrogen bonds of MPC polymer brushes. The element ratios of N and P element contents of PDMS-PMPC were 4.43% and 4.66%, whereas the characteristic peaks of N-1s and P-2s were barely observed on the pristine PDMS. From the XPS spectra of C-1s (Figure 9b), new characteristic peaks were observed at C-O (286 eV) and C=O (288.5 eV) for the PDMS-PMPC substrates. In addition, an additional peak of N-1s spectra (Figure 9c) was found at 402.4 eV, which is the functional group of N^+^(CH_3_)_3_ on the PDMS-PMPC substrate. These results are similar to the FTIR analysis (Figure 8), indicating that the PMPC polymer brushes can grow on the replica of the bionic PDMS surface after atmospheric plasma polymerization.

### 3.6. Antibacterial Adhesion Test

**Figure 10:**
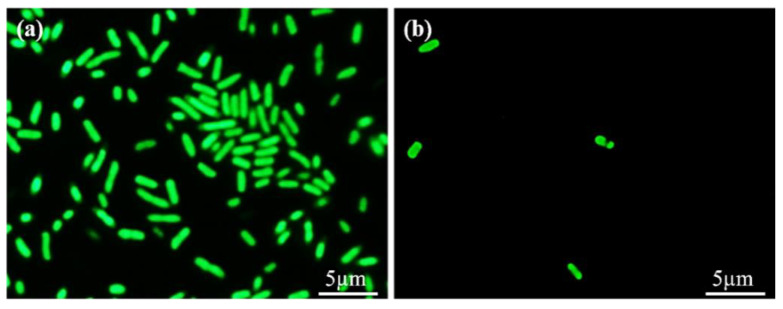
The anti-fouling capability of bacteria (*E. coli*) on the (**a**) pristine PDMS and (**b**) PDMS-PMPC substrate.

The antibacterial effect of PDMS and PDMS-PMPC against bacteria (*E. coli*) was assessed using the bacterial adhesion method. The fluorescence images of bacterial morphology (Figure 10) demonstrate that the bacteria are attached to the sample surface. We noticed a higher number of bacteria on the pristine PDMS substrate (Figure 10a) than on the PMPC-coated PDMS substrate (Figure 10b). This is because the PMPC structure forms the steric hindrance, and the steric hindrance enables the PDMS surface to display antibacterial properties [28].

### 3.7. Anti-Protein Adhesion Test

**Figure 11:**
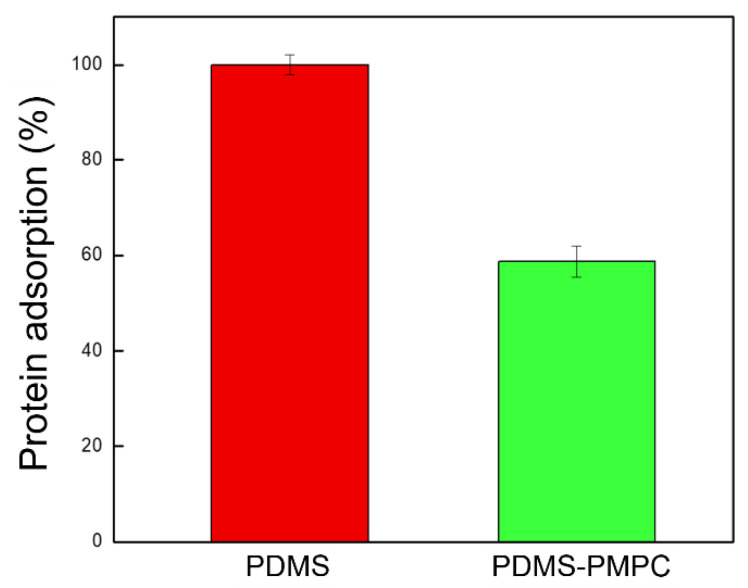
The anti-fouling capability of proteins (human serum albumin, HSA) on the pristine PDMS and PDMS-PMPC substrates (*n* = 3).

The anti-protein adsorption performance of the PDMS and PDSA-PMPC surfaces were quantitatively evaluated by a BCA protein assay (Thermo Fisher, USA). Figure 11 shows the amounts of human serum albumin (HSA), which adhered to the pristine and PMPC-coated PDMS substrates. The adsorbed amount of HSA on the pristine PDMS was 60% higher than the PDMS-PMPC substrate. The PMPC was very effective in preventing the protein from adsorption. This result corresponded with the contact angle. The smaller the water contact angle after PMPC grafting, the greater the anti-fouling capability of proteins [34].

### 3.8. Cells Attachment Test

**Figure 12:**
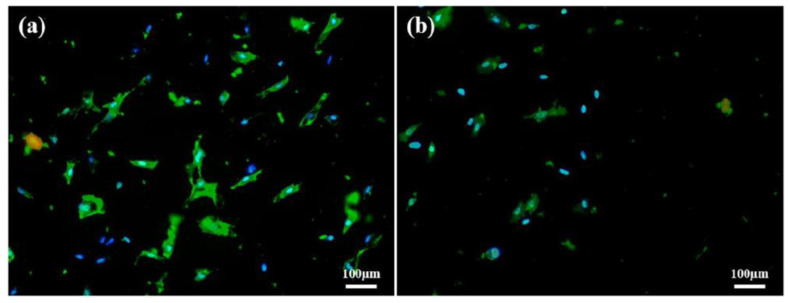
3T3 cells attachment test on the (**a**) pristine PDMS and (**b**) PDMS-PMPC substrates.

The results of 3T3 cells attachment on PDMS and PDMS-PMPC substrate are shown in Figure 12. Although the PDMS surface was hydrophobic, the amount of surface cell adhesion was concentrated and dense (Figure 12a). The PDMS grafted on PMPC (Figure 12b) made it difficult for the cells to attach to the substrate due to the change in surface charge, reduced the number of cells attached, and kept the cell adhesion survival rate.

### 3.9. Biocompatibility Test

**Figure 13:**
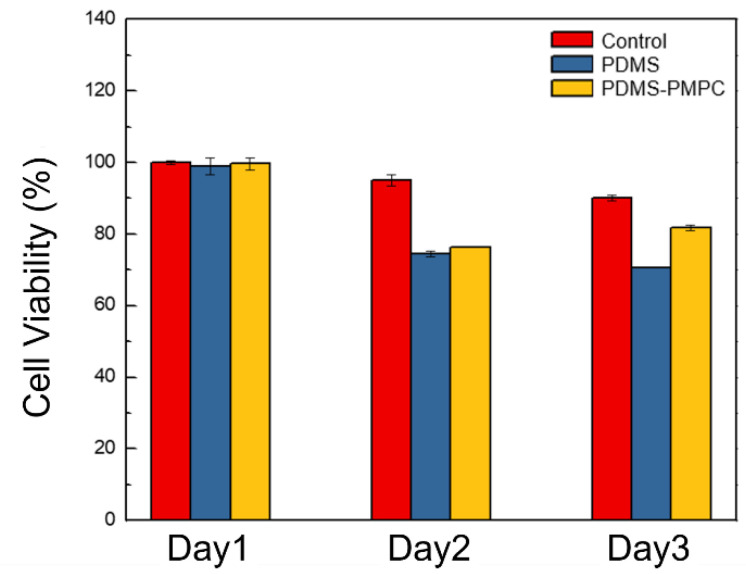
Biocompatibility tests of control, pristine PDMS, and PDMS-PMPC substrates (*n* = 3).

Biocompatibility is defined as the ability of a material to perform with an appropriate host response in a specific application [31]. That is, the material will respond appropriately when it comes into contact with the host. In this study, the biocompatibility of pristine PDMS and PDMS-PMPC was evaluated with the proliferation of NIH 3T3 cells, which are mouse embryonic fibroblasts and have been widely used in biocompatibility tests. Figure 13 shows that 3T3 cells proliferated on the control, PDMS, and PDMS-PMPC substrates at 1–3 days. The results show that cell viability (%) at day 1 was almost the same in the three samples, but pristine PDMS and PDMS-MPC dropped to about 70% at day 2. At day 3, the cell viability (%) of 3T3 cells on the PDMS continued to decline, whereas that on the PDMS-PMPC increased to 85%, similarly with the control group. This demonstrates that PMPC immobilization on the surface can effectively improve biocompatibility at day 3, compared to the pristine PDMS.

### 3.10. One-Way Liquid Transfer Capability

**Figure 14:**
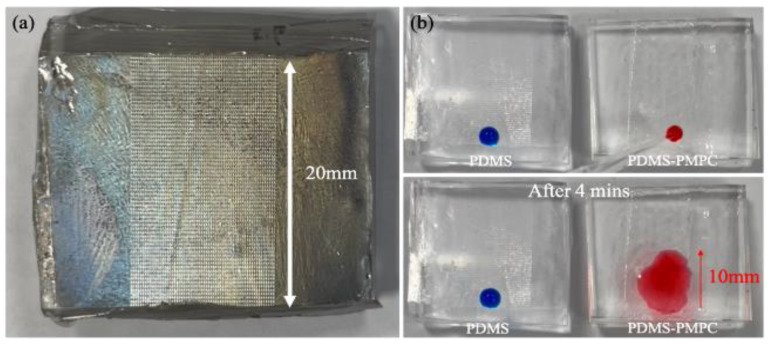
(**a**) The liquid channels (20 mm) of the PDMS-based substrate were used for the channels of the real Nepenthes peristome; (**b**) the stained deionized water (10 μL) was used for the one-way liquid transfer capability (blue water for PDMS and red water for PDMS-PMPC).

The liquid channels (20 mm) were fabricated by imitating channels of the real Nepenthes peristome, as shown in Figure 14a. The stained deionized water (10 μL) was utilized for its one-way liquid transfer capability. The results indicated that the ability of self-driving and unidirectional water delivery was found in the PDMS-PMPC substrate (red-stained water) after 4 min incubation (running distance: ~10 mm), compared with the sticky blue stained water in the PDMS substrate (Figure 14b). The delivery rate of PDMS-PMPC was about 2.5 mm/min, which provides the chromatographic capability of the complex samples. The complex samples could be separated in the chromatographic process (depending on the polarity or molecular weight of the samples), and then detected using the SERS technique.

### 3.11. SERS Detection by Raman Spectroscopy

**Figure 15:**
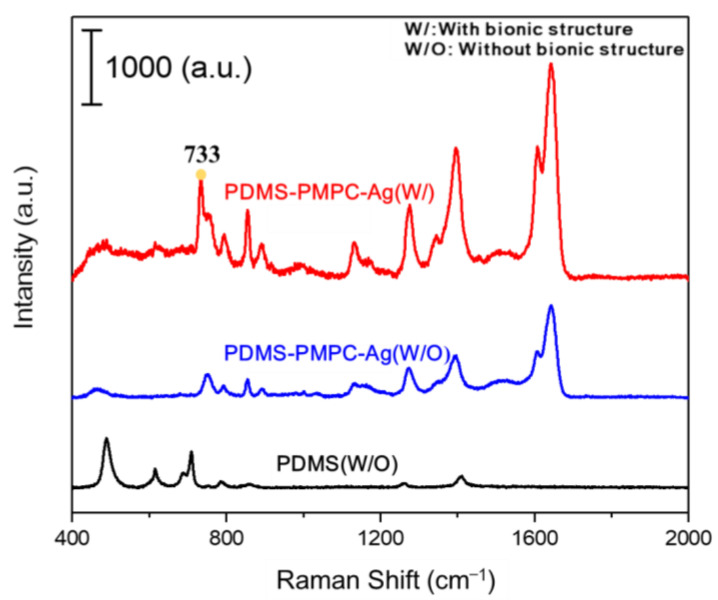
Raman spectrum of PDMS without (W/O) bionic structure, PDMS-PMPC-Ag without (W/O) bionic structure, and PDMS-PMPC-Ag with (W/) bionic structure.

To enable the SERS detection (Figure 15), the replica PDMS-PMPC with a bionic structure was deposited on 5 nm of Ag nanofilm (PDMS-PMPC-Ag (W/)) by thermal evaporation, and pristine PDMS-PMPC with Ag (PDMS-PMPC-Ag (W/O)) deposition was used as the control. The 633 nm laser was used for Raman spectroscopy, and adenine (10^−4^ M) was used as the model biomolecules analytes. The results indicated a characteristic peak of adenine at 733 cm^−1^, and the SERS intensity of PDMS-PMPC-Ag (W/) was ~4 times stronger than that of PDMS-PMPC-Ag (W/O), which indicated that multi-reflection by the 3D bionic structure enhanced SERS intensity. The pristine PDMS without Ag deposition and bionic structure (PDMS (W/O)) is displayed as a black line in the background.

## 4. Conclusions

In this study, we successfully fabricated a bionic Nepenthes peristome-like structure by photolithography with SU-8 photoresist. Furthermore, the bionic structure was replicated by a flexible PDMS substrate and then immobilized with MPC polymer brushes by an atmospheric plasma treatment to improve its hydrophilicity, antibacterial attachment, anti-protein adsorption, and biocompatibility. The self-driving capability of SERS detection and the one-way liquid transfer were demonstrated on the replica of the bionic PDMS-PMPC substrate. A benefit of the Nepenthes peristome-like structure with PMPC modification was that the delivery rate was about 2.5 mm/min, unlike the sticky stained water on the unmodified PMPC surface. This can provide the chromatographic capability to separate complex samples, and further detect them by Raman spectroscopy. The flexible and multi-functional SERS chips could potentially be applied in a wearable device for biomedical and environmental detection.
